# Insulin receptor signaling in the development of neuronal structure and function

**DOI:** 10.1186/1749-8104-5-7

**Published:** 2010-03-15

**Authors:** Shu-Ling Chiu, Hollis T Cline

**Affiliations:** 1 Watson School of Biological Sciences and Cold Spring Harbor Laboratory, Cold Spring Harbor, NY 11724, USA; 2 Departments of Cell Biology and Chemical Physiology, The Scripps Research Institute, 10550 North Torrey Pines Road, La Jolla, CA 92037, USA

## Abstract

Sensory experience plays a crucial role in regulating neuronal shape and in developing synaptic contacts during brain formation. These features are required for a neuron to receive, integrate, and transmit signals within the neuronal network so that animals can adapt to the constant changing environment. Insulin receptor signaling, which has been extensively studied in peripheral organ systems such as liver, muscle and adipocyte, has recently been shown to play important roles in the central nervous system. Here we review the current understanding of the underlying mechanisms that regulate structural and functional aspects of circuit development, particularly with respect to the role of insulin receptor signaling in synaptic function and the development of dendritic arbor morphology. The potential link between insulin receptor signaling malfunction and neurological disorders will also be discussed.

## Introduction

The human brain is made up of billions of neurons assembled into sophisticated circuits. Information received from sensory neurons is processed by neurons within distinct circuits to generate specific functional outputs, including cognitive decisions and behavior. A fascinating problem is how these huge numbers of neurons establish precise connections to assemble complex circuits during development. The neuron, the functional unit of the brain circuit, is a highly specialized cell composed of the cell body, the dendrite and the axon. The structure of the dendrite determines where and how an individual neuron can receive and integrate information from afferent neurons, whereas the morphology of the axon determines where processed information is sent to efferent neurons. Sites of contacts between the axon and dendrite, or synapses, mediate communication between neurons for proper information flow within the neuronal circuit. We will first review the current understanding of cellular and molecular mechanisms underlying synapse and dendrite development, then focus on recent evidence suggesting a function for insulin receptor signaling in circuit function and pathological brain diseases.

## Synapse and dendrite development

### Synapse development

The number of synaptic contacts and the efficacy of synaptic transmission in the brain are dynamic throughout development and adulthood [1-3]. These dynamics are crucial for neurons to optimize connections in brain circuits during development. Synaptic plasticity is also important to optimize neuronal function in adults, for example, to adapt to our changing environment and to allow memories to form. Synapse development is a series of distinct processes, including synapse formation, synapse maturation and synapse maintenance. The mechanisms that regulate each of these processes are just starting to be unraveled.

#### Synapse formation

**Figure 1:**
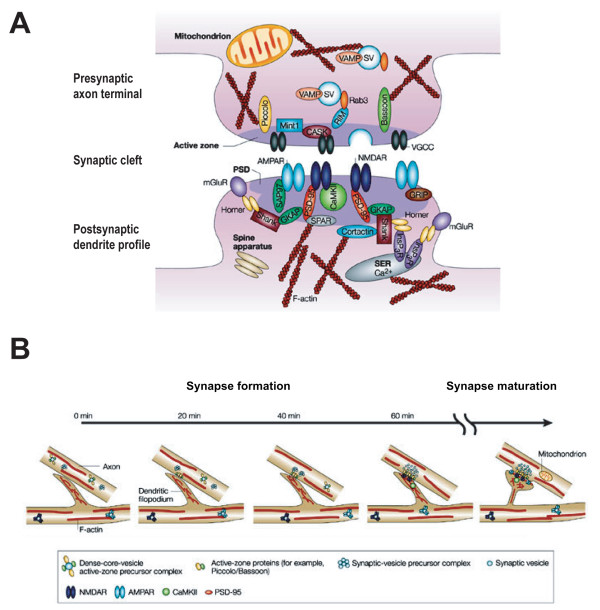
**Schematic diagram of an excitatory synapse and the temporal sequence of synapse formation and maturation**. **(A) **Synapses are specialized junctions between neurons composed of complex membrane and proteins. A synapse can be divided structurally into three parts: a presynaptic axon terminal packed with synaptic vesicles (SV) and release machinery, a synaptic cleft, and a postsynaptic dendritic counterpart filled with neurotransmitter receptors, scaffold proteins and signaling machinery. **(B) **Synapse formation is initiated by the contact between dendrites and axons, followed by the recruitment of presynaptic and postsynaptic specializations. Increases in synapse size and synaptic strength by accumulation of AMPA receptors at synapses are characteristics of synapse maturation. AMPAR, α-amino-3-hydroxy-5-methyl-4-isoxazole propionic acid receptor; CaMKII, Calcium calmodulin dependent kinase type II; CASK, calcium calmodulin-dependent serine kinase; GKAP, guanylate kinase-associated protein; GRIP, glutamate receptor-interacting protein; InsP_3_R, inositol triphosphate receptor; mGluR, metabotropic glutamate receptor; NMDAR, N-methyl-D-aspartate receptor; PSD, postsynaptic density; PSD-95, postsynaptic density protein-95; RIM, Rab3-interacting molecule; SAP, synapse-associated protein; SER, smooth endoplasmic reticulum; SPAR, spine-associated Rap GTPase activating protein; VAMP, vesicle-associated membrane protein; VGCC, voltage-gated calcium channel. (Adapted and modified from [4]).

Synapses are specialized junctions between neurons where the presynaptic axon terminal is packed with synaptic vesicles and vesicle release machinery and the postsynaptic dendritic specialization contains transmembrane neurotransmitter receptors, scaffold proteins and signaling machinery (Figure 1A; see [4] for detailed review). Time-lapse imaging in both *in vivo *and *in vitro *preparations revealed that the temporal sequence of synapse formation is quite rapid. The first step involves the contact between dendrites and axons, which likely occurs by adhesive mechanisms. Second, the presynaptic specialization assembles quickly at sites of contact [5,6]. In fact, it is thought that components of the presynaptic specialization are present in axons before synaptogenesis as packets of vesicle proteins and components of the active zone proteins [7,8], Finally, the postsynaptic specialization, including the proteins postsynaptic density-95 (PSD-95), and neurotransmitter receptors, including N-methyl-D-aspartate (NMDA) receptors, are thought to arrive somewhat later during synapse formation (Figure 1B) [9]. Although the assembly of synapses is a complex process, recent work has identified several molecules that are important in different steps of synapse formation [7,10]. For example, molecules that are present in gradients within target regions, such as ephrins, play an important role in directing axons and dendrites to the correct brain regions [11,12]. Adhesion molecules, such as cadherin, are thought to be important in establishing of the initial axodendritic contacts [13,14]. Some transsynaptic molecules, such as neuroligin and neurexin, are crucial in bidirectional signaling and the recruitment of both pre- and postsynaptic proteins to new synapses [15,16]. In addition to molecular players, neuronal activity appears to be another key regulator in the formation of nascent synapses [17-20].

#### Synapse maturation

Synapse maturation is characterized by an increase in the morphological size and transmission strength of the synapse, which includes changes in both the presynaptic axon terminal and the postsynaptic dendrite. From the presynaptic point of view, a prominent ultrastructural feature of synaptic maturation is the increase in the number of synaptic vesicles per terminal [21-23], which likely contributes to the increase in probability of transmitter release in mature synapses [17]. Transmission at immature glutamatergic synapses is mainly mediated by NMDA receptors, which shift their kinetics by replacing NMDA receptor subunit 2B-containing receptors with NMDA receptor subunit 2A-containing receptors [24,25]. These immature synapses can be silent or have low synaptic strength at resting membrane potentials because of voltage-dependent magnesium blockade of the NMDA receptor. As the synapses mature, α-amino-3-hydroxy-5-methyl-4-isoxazole propionic acid (AMPA) receptors are recruited to the postsynaptic membrane, and in addition to NMDA receptors, provide fully functional glutamatergic synaptic transmission [26-29]. Neuronal activity reportedly induces synapse maturation by promoting the incorporation of NMDA receptor subunit 2A-containing NMDA receptors into synaptic sites. Furthermore, activity recruits AMPA receptors to the postsynaptic site to activate silent synapses and increase the strength of synaptic transmission (Figure 1B) [4,26,27].

#### Synapse maintenance or synapse elimination

The precise connectivity required for circuit function relies not only on the formation of new contacts but also the maintenance of the correct synapses. In fact, the density of synapses formed at early stages of development is far greater than the density retained at later stages, indicating that only selective synapses are stabilized and maintained during development [30]. The importance of synapse maintenance is well documented at the neuromuscular junction, where each muscle fiber is temporarily innervated by multiple motor axons but only one input becomes stabilized while others are eliminated [31,32]. A reduction in synapse density has also been demonstrated in various regions of the central nervous system (CNS) [33-37], suggesting synapse elimination could be a common process for refining the brain circuits during development. For instance, climbing fiber to Purkinje cell synapses in cerebellum undergo synapse elimination at early postnatal ages in mammals. Although the detailed mechanisms regulating synapse elimination and maintenance remain largely unknown, neuronal activity appears to contribute to the maintenance of correct synapses while weaker synapses are usually eliminated [34,38,39]. Additionally, molecular players such as insulin-like growth factor (IGF)-2, complement protein 1q, major histocompatibility complex protein, protein kinase Cγ, metabotropic glutamate receptor subtype 1, and glutamate receptor (GluR) delta 2 subunit reportedly regulate synapse maintenance or elimination [40-45].

### Dendrite development

The architecture of the dendritic arbor contributes to the precise patterning of synaptic connections required for normal circuit function. Dendritic structure not only determines which axons are potential presynaptic partners, but also determines how the inputs are integrated. The marriage of single cell labeling and *in vivo *time-lapse imaging has made it possible to explore the cellular mechanisms underlying dendritic development [19,20,46,47]. Advances in microscopy, cell biology and molecular genetic methods have paved the way for significant advances in our understanding of the mechanisms behind the molecular and activity-dependent regulation of dendrite development.

#### Cellular mechanisms

**Figure 2:**
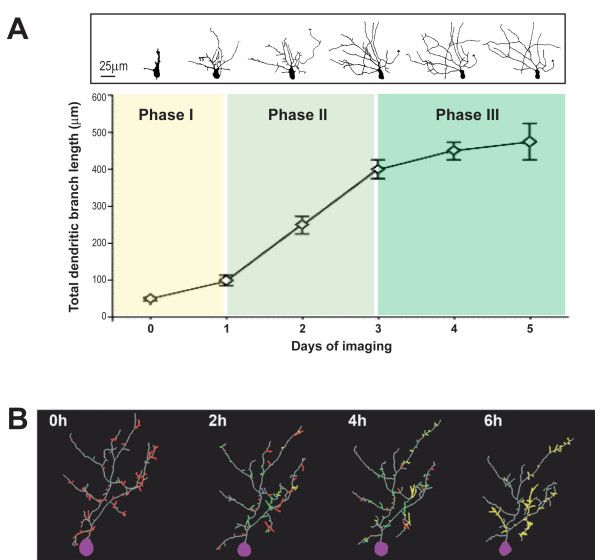
**Dendritic arbor growth and dynamics**. **(A) **Dendrite development can be divided into three phases: phase I, the cell differentiates and extends an axon with little elaboration of the dendritic arbor; phase II, the dendritic arbor grows rapidly; phase III, the dendritic arbor grows slowly and appears stable. Reconstruction of the same tectal neuron imaged *in vivo *daily for 5 days is shown on top. (Adapted and modified from [19,48].) **(B) **Dendritic growth is highly dynamic as revealed by time-lapse imaging at 2-hour intervals over a 6-hour imaging period. Stable branches, retracted branches, transient branches, and added branches are shown in gray, red, green and yellow, respectively. Cell bodies are shown in purple. (Adapted from [52].)

Imaging optic tectal neurons *in vivo *in *Xenopus *tadpoles showed that dendritic arbor elaboration goes through distinct phases (Figure 2A) [19,48]. Many newly differentiated neurons first undergo axonogenesis with only little elaboration of the dendritic arbor. About one day after evidence of morphological differentiation, neurons go into a rapid dendritic arbor growth phase for a few days until they enter the third phase, characterized by a slower dendritic arbor growth rate and more stable dendritic arbors. During the rapid dendritic arbor growth period, one might think that the growth of the dendritic arbor can be easily achieved by continuously lengthening pre-existing dendrites and sprouting new dendritic branches; however, time-lapse imaging at intervals of minutes to hours reveals that dendritic growth is highly dynamic, consisting of not only branch addition and extension, but also retraction and loss of dendritic branches (Figure 2B) [18,49-52]. It is worth noting that these dynamics in dendritic morphogenesis persist in mature neurons when their overall structure is stable, although at a slower rate [20,53-55]. Therefore, it is very likely that mechanisms that regulate dendritic dynamics early during development may also play a role in dendritic plasticity later in life.

#### Molecular mechanisms

Mechanisms that regulate cytoskeleton architecture play a crucial role in shaping dendritic arbors because the cytoskeleton provides the fundamental support of the dendritic structure. Filopodia are thin, highly motile actin-based protrusions and some of them are transformed into more stable microtubule-based dendritic branches. The Rho family of small GTPases, including Rac, RhoA, and Cdc42, regulate the rearrangement of cytoskeleton and participate in distinct aspects of dendrite morphogenesis [56,57]. For example, Rac and Cdc42 activity promote dendritic arbor dynamics by increasing the rate of actin polymerization, whereas increased RhoA activity inhibits dendritic arbor growth in *Xenopus *tectal neurons [57]. Consistently, several guanine exchange factors that activate Rac, such as Tiam1 [58] and STEF [14,59,60], have been shown to regulate neurite formation whereas Rho-specific guanine exchange factors, such as KIAA0380 [14], and Rho-specific GTPase activating proteins, such as p190 RhoGAP [61], which activate or inactivate Rho, respectively, have been shown to regulate neurite retraction [14,59,60]*in vitro*. Interestingly, there is considerable crosstalk among these Rho GTPases. RhoA activity was increased by Rac activation and Cdc42 inhibition, whereas Rac was inhibited by activation of Rho in *Xenopus *tectal neurons *in vivo *[62]. This tight cross-regulation of Rho GTPases seems to work together to determine the structure of the dendritic tree. What controls the activity of Rho GTPases is a key question to understand the underlying mechanisms in dendritic morphogenesis.

In the *Xenopus *visual system visual activity promotes dendritic arbor growth through mechanisms that require both glutamate receptor activity and Rho GTPase activity in *Xenopus *tectal neurons [63]. Accordingly, the working hypothesis is that glutamate receptor activity promotes dendritic growth by elevating Rac and Cdc42 activities, leading to increased branch dynamics, and concurrently decreasing RhoA activity to relieve its inhibition on branch extension [63]. In addition to Rho GTPases, several other molecular mechanisms, including signaling through neurotrophins [64,65], CPG15 [66] and calcium calmodulin dependent kinase type II [67], or local protein synthesis, mediated by cytoplasmic polyadenylation [47], have been shown to regulate dendritic arbor development in an activity-dependent manner. These data suggest that dendritic arbor growth is organized by signals from their surrounding environment.

#### Synaptic function and dendritic development

During circuit development, the increase in synapse number and synaptic strength occur concurrently with the elaboration of dendritic arbors, suggesting a coordinated regulation of synaptic function and dendritic development. Almost two decades ago, Vaughn first proposed the 'synaptotrophic hypothesis', which states that the stabilization of synapses might stabilize the dendritic branches and thereby explain the coordinated development of synapses and dendritic arbors [68]. Recent research has provided new supporting evidence for this hypothesis. Adhesion molecules, which play important roles in the initial assembly and stabilization of the synapses, regulate dendritic arbor development in mammals and flies [69,70]. Moreover, live imaging of synapse formation and dendrite growth in zebrafish showed that the presence of the synapses associated with the stabilization of terminal dendrites [71]. On the other hand, blockade of synaptic transmission or synapse maturation reduces dendritic arbor elaboration and blocks activity-dependent dendritic growth in *Xenopus *[18,50,52,63,72]. It is interesting to note that decreasing GABAergic transmission also changes the pattern of dendritic arbor growth and blocks visual experience-dependent structural plasticity [73]. These data suggest that synaptic contacts and synaptic transmission regulate the growth and elaboration of complex dendritic arbors in sculpting circuit function during development.

## The insulin receptor

The insulin receptor is a receptor tyrosine kinase well studied with regard to its function in the regulation of peripheral glucose metabolism. Although expression of the insulin receptor in the brain was discovered decades ago [74,75], insulin receptor function in this classic 'insulin-insensitive' organ remains largely unknown. Recent studies in neuronal cell culture suggest that insulin receptor signaling regulates several neuronal functions, including spine density and neurite growth [76,77]; however, the role of insulin receptor signaling in controlling structure and function of CNS circuit development has not yet been widely explored *in vivo*.

### Structure and signaling of the insulin receptor in peripheral tissues

**Figure 3:**
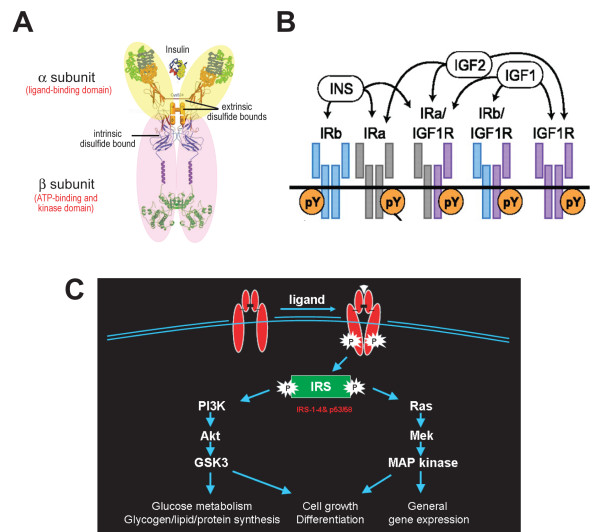
**Insulin receptor structure and signaling**. **(A) **Insulin receptor monomer, composed of an α (yellow) and β subunit (pink) bridged by an intrinsic disulfide bond, which dimerizes with another insulin receptor monomer through extrinsic disulfide bonds to form a functional receptor. (Adapted and modified from [80].) **(B) **Ligand selectivity of the insulin receptor homodimer or heterodimer with the insulin-like growth factor (IGF)-1 receptor. Note that the homodimer of the splice variant IRa, the predominant form of inslulin receptor in the brain, binds specifically to insulin (INS), whereas the heterodimer with the IGF-1 receptor binds to not only INS but also IGF-1 and IGF-2. (Adapted and modified from [86].) **(C) **Insulin receptor signaling initiated by ligand binding activates tyrosine autophosphorylation in the β subunit, which stimulates two major downstream pathways, the phosphoinositide-3 kinase (PI3K)/Akt and Ras/mitogen-activated protein kinase (MAPK) cascades, through insulin receptor substrates (IRSs) and results in a diverse series of cellular processes in peripheral tissues. (Modified from [88].) GSK3, glycogen synthase kinase 3.

The insulin receptor was first found as a homodimer, with extrinsic disulfide bonds to generate the functional receptor. Each monomer of the insulin receptor is composed of one α and one β subunit bridged by an intrinsic disulfide bond [78,79]. The 135-kDa α subunit is the extracellular ligand binding portion, whereas the 95-kDa β subunit consists of an extracellular, a single transmembrane, and an intracellular kinase domain. Ligand binding to the α subunits activates the intrinsic kinase activity located in the β subunits and subsequently initiates a cascade of phosphorylation events that leads to different biological functions (Figure 3A) [80].

Crystal structures of the unphosphorylated and phosphorylated kinase domains of the insulin receptor have provided detailed information on how insulin receptor kinase activity is initiated. The kinase domain is composed of two lobes, the amino- and carboxy-terminal lobes, with an activation loop in between. In the unphosphorylated state, the activation loop traverses the cleft between two lobes such that both ATP binding and protein substrate-binding sites are blocked. More specifically, while residues in the beginning of the activation loop restrict the access of ATP to its binding sites on the insulin receptor, tyrosine 1162, one of the three phosphorylation sites in the activation loop, binds to the active site and competes with the kinase substrates [81]. Autophosphorylation of tyrosine 1158, 1162 and 1163 in the activation loop of the kinase domain causes rearrangement of the activation loop and reorientation of the amino- and carboxy-terminal lobes of the kinase, which is necessary for productive ATP binding. Tyrosine 1163 is the key phosphotyrosine in stabilizing the conformation of this phosphorylated activation loop, whereas tyrosine 1158 is accessible for interaction with downstream signaling proteins [81]. The knowledge of insulin receptor structure not only provides valuable understanding on how insulin receptor signaling is transduced but also allows functional analysis following the generation of various mutants of the putative ATP binding site or phosphorylation sites [82-85].

Unlike other receptor tyrosine kinases, most functions of the insulin receptor require accessory molecules known as insulin receptor substrates (IRSs) - for example IRS1-4- to engage multiple downstream signaling [86,87]. Two major cellular signaling pathways, phosphoinositide-3 kinase (PI3K)/Akt and the Ras/mitogen-activated protein kinase (MAPK) pathways, can be activated by the kinase activity of insulin receptor. These cascades regulate diverse cellular processes, such as gene expression, protein synthesis, and vesicle trafficking, which result in the regulation of glucose, lipid and protein metabolism, cell growth and differentiation (Figure 3C) [88,89].

### Diversity of the insulin receptor

Although the insulin receptor is encoded by one single gene, various processes give rise to considerable diversity in its protein structure and function. First, alternative splicing produces two isoforms of insulin receptor, IRa, an exon-11 lacking form, and IRb, an exon-11 containing form in a tissue-specific manner. Moreover, post-translational glycosylation contributes to different modifications of these receptors in different cell types or tissues [90]. Furthermore, assembly of hybrids between different isoforms and heterodimers with homologous IGF-1 receptor results in further diversity [91,92]. Although different ligand binding affinity and selectivity have been reported for insulin receptors, the physiological significance of the splice variance, post-translational modification and homo- or hetero-dimerization between different insulin receptors and IGF-1 receptor remain largely unknown (Figure 3B) [86,93]. Neurons have mainly the IRa isoform with less glycosylation compared to glial cells or peripheral tissues [94]. The different properties of neuronal insulin receptors might suggest different roles of insulin receptors in the CNS. Interestingly, IRα binds insulin or IGF-2 with comparable affinity [95] and hybrids of IRa with the IGF-1 receptor binds IGF-1, IGF-2 and insulin with similar affinity [86,93]. Taken together, these data suggest that, in addition to insulin, IGF-1 and IGF-2 are potential ligands for the insulin receptor in the brain. The capability of neuronal insulin receptors to interact with various ligands suggests that insulin receptors may play versatile functions in the CNS.

**Figure 4:**
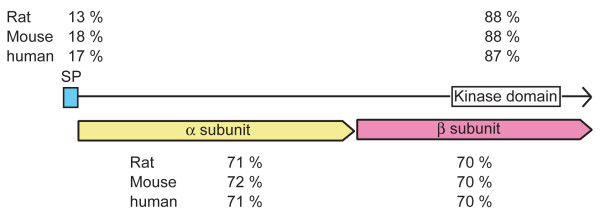
**Protein sequence alignment of *X. laevis *insulin receptor IR1 with different species**. Amino acid sequence derived from *Xenopus *IR1 was aligned with rat, mouse and human insulin receptor protein sequences with the ClustalW algorithm. Schematic drawing of the alignment identities (percentages) in different functional domains of insulin receptor. Note that the predicated *Xenopus *kinase domain shares the highest identity with other species compared to other domains.

In contrast to other species, *Xenopus laevis *has two insulin receptor genes, which we isolated from brain cDNA libraries. At the nucleotide level, these two *Xenopus *brain insulin receptors are highly similar to each other (93.6% identity) and are splice variants homologous to a human brain isoform of insulin receptor lacking exon-11 [96]. At the amino acid level, the corresponding peptides of these two *Xenopus *insulin receptors, termed IR1 and IR2, share overall 95% identity and 97% similarity. Since only one insulin receptor gene has been reported in human or other vertebrates, the two copies of insulin receptor genes potentially result from the tetraploid nature of the *X. laevis *genome [97]. Alignment of the amino acid sequence of *IR1*, the more abundant *Xenopus *insulin receptor, with other species showed that the *Xenopus *insulin receptor shares overall identities of 70%, 69% and 69% with those of human, rat and mouse, respectively (Figure 4). Detailed alignments of different domains of insulin receptor further revealed that the kinase domain of the *Xenopus *insulin receptor shares the highest identity of 87 to 88% with that of human, rat and mouse compared to other regions (Figure 4). In addition, the potential ATP binding site and phosphorylation sites on the activation loop of the kinase domain [81] are remarkably identical to those of human, rat and mouse, suggesting that these amino acids may play a functional role in insulin receptor action and thus are well conserved in evolution. This also suggests that it is suitable to be mutated for studying insulin receptor function *in vivo*.

### Expression pattern of insulin receptor in the brain

The insulin receptor is distributed in a widespread, but selective, pattern in the brain, including olfactory bulb, cerebral cortex, hypothalamus, hippocampus and cerebellum as reported in rodents [74,75,90]. The expression level of the insulin receptor is developmentally regulated, being higher at early stages and lower in the adult. At the cellular level, the insulin receptor is enriched in neurons compared to glia [74]. Subcellularly, the insulin receptor is a component of synapses, where it concentrates at the postsynaptic density (PSD) in cultured hippocampal neurons [98]. These data together suggest that the insulin receptor is in the right place at the right time to regulate the initial neuronal development by regulating synaptic function in the CNS. Although the IGF-1 receptor, which can dimerize with the insulin receptor and affect its ligand affinity and specificity, as mentioned previously, shows a similar distribution in the brain as the insulin receptor, it also exhibits a distinct expression pattern compared to the insulin receptor when examined in detail locally [99]. For example, both receptors are highly expressed in hippocampus; however, insulin receptor mRNA is more abundant in the CA1 region whereas IGF-1 receptor mRNA is greater in the CA3 region, implying that insulin/IGF-1 receptor signaling may play distinct roles in subregions of the hippocampus.

### Function of insulin receptor in circuit development and plasticity

Brain insulin receptor signaling reportedly plays diverse roles in the CNS, including regulation of synaptic plasticity [100-106], dendritic outgrowth [77,107], and involvement in neuronal survival [108,109], life span [110-114], learning and memory [115-117], and neurological disorders [118-121]. A role for insulin receptor signaling in synaptic function and dendritic morphogenesis, therefore, makes it a potential regulator of circuit development and circuit function.

#### Synaptic function

Recent work suggests insulin receptor signaling is involved in postsynaptic neurotransmitter receptor trafficking. For excitatory receptors, insulin accelerates clathrin-dependent endocytosis of GluR2 subunit-containing AMPA receptors and contributes to long-term depression [100,102,122-124]. In contrast, insulin reportedly accelerates GluR1 subunit-containing AMPA receptor insertion into the membrane in a GluR1 subunit-dependent manner in cultured hippocampal neurons [104]. Therefore, the physiological significance of insulin receptor signaling in AMPA receptor-mediated transmission is somewhat controversial and needs to be further studied *in vivo*. Moreover, insulin promotes the delivery of NMDA receptors to the cell surface by exocytosis in *Xenopus *oocytes expressing recombinant NMDA receptor [105]. For inhibitory receptors, insulin rapidly recruits type A γ-aminobutyric acid (GABA_A_) receptors to the postsynaptic membrane in cultured hippocampal neurons [106]. These data suggest that insulin receptor signaling is capable of regulating both excitatory and inhibitory synaptic transmission in the CNS. In addition, brief incubation of insulin results in increased protein synthesis of PSD-95, a dendritic scaffolding protein that associates neurotransmitter receptors and cytoskeletal elements at synapses in hippocampal slices and synaptosomes [125], also suggesting that insulin receptor signaling can potentially regulate structural aspects of synaptic function, synaptogenesis and synapse maturation.

**Figure 5:**
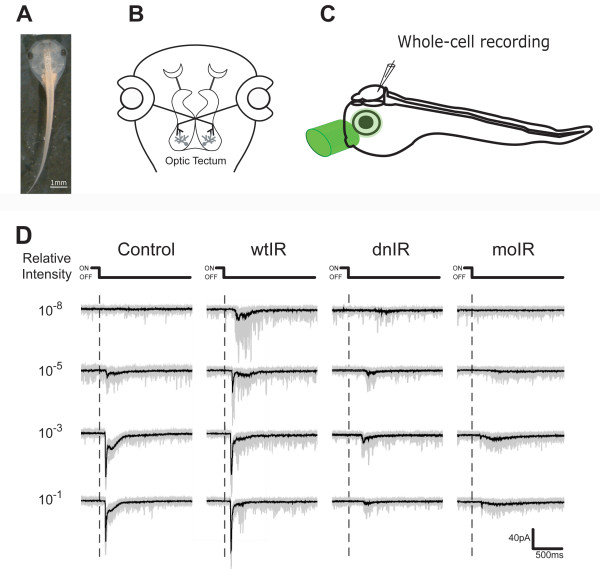
**Recording of visual responses in an intact animal**. **(A) ***X. laevis *tadpole. **(B) **Diagram of the *Xenopus *visual circuit. Optic tectal neurons receive direct visual input from retinal ganglion cells of the eye. **(C) **Experimental setup. Visual stimulation was delivered with a green LED pigtailed to an optic fiber that illuminates the entire eye for whole field stimulation. The tadpole brain was cut along the midline to expose the cell bodies. Visual responses from tectal neurons contralateral to the stimulated eye were recorded by whole-cell recording. **(D) **Visual responses of tectal neurons. Tectal neurons respond to transient light intensity change. The OFF responses induced by light off is normally bigger in amplitude and longer in duration than ON responses induced by light on. Superimposition of 20 consecutive responses (gray) and the averaged trace (black) are shown. Adapted from [84]. dnIR, dominant negative insulin receptor; moIR, morpholino-mediated knockdown of insulin receptor; wtIR, wild-type insulin receptor.

Recently, our laboratory provided direct *in vivo *evidence for the function of insulin receptor signaling in both the structure and function of brain circuit development in the visual system of live *Xenopus *tadpoles [84]. The retinotectal circuit of *Xenopus *(see schematic in Figure 5), in which tectal neurons receive direct visual input from the retinal ganglion cells in the eye [126], is a powerful experimental system to study both structural [47,52,63,73] and functional plasticity [17,36,47,73,127-130]*in vivo*. By taking advantage of the *Xenopus *visual circuit as an *in vivo *experimental system amenable to molecular manipulation, electrophysiology and a variety of imaging methods, we showed that the insulin receptor is required for optic tectal neurons to receive normal levels of visual input within the retinotectal circuit [84]. Reduced insulin receptor phosphorylation by ectopic expression of dominant negative insulin receptor (dnIR), which contains a point mutation to abolish insulin receptor binding to ATP, or decrease insulin receptor protein by morpholino-mediated knockdown in tectal neurons, severely decreases their glutamatergic synaptic input and reduces their responses to natural light stimuli (Figure 5). Few studies have made a direct comparison between the effects of protein knockdown and dominant negative interference with signaling. It is interesting to note that decreasing insulin receptor signaling either by expression of a dominant negative receptor or by morpholino-mediated knockdown leads to a comparable magnitude of functional impairment in visual system processing, suggesting that the presence of the protein itself does not play a role in visual system development independent of its kinase-dependent signaling.

#### Dendritic morphogenesis

Several molecules downstream of the insulin receptor, including both the Ras/MAPK and PI3K/Akt/mammalian target of rapamycin (mTOR) pathways, have been implicated in excitatory synaptic connectivity as well as dendritic structure [131,132]. IRSp53, a novel insulin receptor substrate enriched in the brain, where it localizes to synapses as a component of the PSD [98], is particularly interesting. Structural analysis predicted that IRSp53 contains several protein-protein interaction domains, including an amino-terminal F-actin bundling domain [133,134], a central Cdc42/Rac interactive binding (CRIB) motif [135], a Src homology region 3 (SH3) domain [76,136,137], a proline rich SH3-binding domain [136], a proline-rich WW-binding motif [136], and a carboxy-terminal postsynaptic density-95/discs large/zona occudens-1 (PDZ) domain [76,138]. Biochemical studies showed that it directly interacts with PSD scaffold proteins, Shank and PSD-95 [76,137-139], small GTPases such as Rac and Cdc42 [77,139-141], and actin regulators such as WAVE2 and Mena [140,141]. These data together suggest a link between insulin receptor signaling and the structural stabilization of excitatory synaptic contacts through the association of synaptic scaffolding proteins and the cytoskeleton. In fact, these ideas were further supported by the findings that overexpression of IRSp53 can increase spine density in cultured hippocampal neurons [76] and induce filopodium formation and neurite outgrowth in N1E-115 neuroblastoma cells [77,142], whereas RNA interference knockdown of IRSp53 protein decreases spine density and alters spine morphogenesis [76]. Another line of evidence supporting the idea that insulin receptor plays a role in dendritic arbor development comes from transgenic mice lacking IGF-1, a potential ligand for insulin receptor and IGF-1 receptor heterodimer receptors in the brain. Pyramidal neurons from the IGF-1 null mice showed significant reduction in dendritic arbor length and complexity as well as spine density [107].

#### Experience-dependent dendritic plasticity

Activity shapes synaptic connectivity and dendritic morphogenesis in the CNS, particularly in sensory regions. Interestingly, insulin is released from neurons upon depolarization [143,144] and IRSp53 translocates to synapses in response to activity [145], suggesting that insulin receptor signaling may increase in an activity-dependent manner. Consistent with this idea, we have shown recently that insulin receptor signaling plays an important role in visual experience-dependent structural plasticity [84]. More specifically, enhanced visual stimulation normally induces tectal neurons to increase their rate of dendritic growth by increasing branch length extension and branch tip stabilization. In the absence of insulin receptor signaling, however, more branches shorten and more branches are lost during the period of visual stimulation.

#### Insulin receptor signaling and synaptic structure

**Figure 6:**
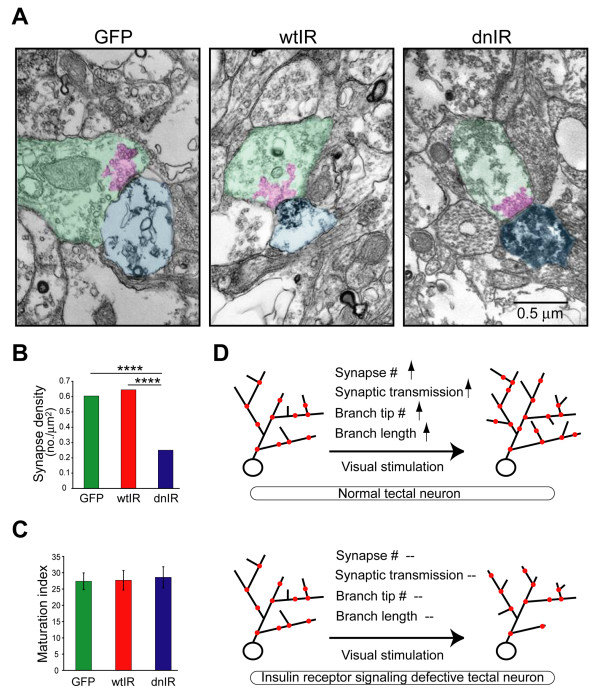
**Insulin receptor signaling regulates synapse numbers**. **(A) **Electron micrographs show ultrastructural morphology of synaptic terminals that contact green fluorescent protein (GFP)-, wild-type insulin receptor (wtIR)- and dominant negative insulin receptor (dnIR)-expressing dendrites. The postsynaptic area, presynaptic area and the clustered synaptic vesicle are highlighted in light blue, green, and pink, respectively. **(B) **dnIR-expressing dendrites receive significantly fewer synaptic contacts compared to GFP- and wtIR-transfected dendrites. **(C) **Synapses that contact GFP-, wtIR- and dnIR-expressing dendrites show comparable ultrastructural synaptic maturity, determined by the area occupied by clustered synaptic vesicles relative to the area of the presynaptic terminal. **(D) **Schematic cartoon showing that normal tectal neurons increase total synapse number and, therefore, synaptic transmission, branch stabilization and extension in response to enhanced visual stimulation, whereas tectal neurons expressing dnIR do not increase synapse number and fail to increase synaptic function and dendritic structural plasticity.

As mentioned earlier, reduced insulin receptor protein and signaling in *Xenopus *visual system showed that insulin receptor signaling is required for optic tectal neurons to receive proper glutamatergic synaptic input and undergo activity-dependent dendritic arbor growth. To probe the role of insulin receptor signaling in developmental plasticity of the glutamatergic synapse, we examined the spontaneous AMPA receptor-mediated miniature excitatory postsynaptic currents (mEPSC) in dnIR-expressing neurons. We found that they have much lower mEPSC frequency but equivalent amplitude compared to controls, indicating that either presynaptic vesicle release probability or synapse number is reduced in dnIR-expressing neurons. Because the paired-pulsed ratio with retinal ganglion cell axon stimulation in dnIR-expressing neurons did not change, it is unlikely that the lower mEPSC frequency in dnIR-expressing neurons is due to low probability of release. To test whether synaptic contacts onto dnIR-expressing tectal neurons were changed in dnIR-expressing neurons, we used electron microscopy to estimate synapse density on tectal neurons. This methodology gives both definite identification of synaptic contacts onto transfected neurons and ultrastructural information about both pre- and post-synaptic profiles [23]. We estimated synapse density by measuring the number of green fluorescent protein (GFP)-labeled synapses normalized to the total area of GFP-labeled dendritic profiles and found that dnIR-expressing dendrites had less than half of the synapse density of GFP-labeled neuron controls, although no changes in other ultrastructural features or synapse maturation were observed (Figures 6A-C) [84]. These observations, according to both electrophysiological and ultrastructural data, together with decreased dendritic plasticity in dnIR-expressing neurons, suggest that insulin receptor signaling maintains both synaptic contacts and the branches they sit on, which in turn promotes dendritic branch elaboration with visual experience.

Our observations are consistent with the synaptotrophic hypothesis, which states that the formation and maintenance of synapses promote the stabilization of dendritic branches and that dendritic arbor growth correlates positively with the number and strength of synapses [18]. In the optic tectum of *Xenopus*, visual experience increases dendritic arbor growth rate, retinotectal synaptogenesis and retinotectal synaptic strength [17,52,63,128,146]. Similarly, in zebrafish, synapses appear to stabilize growing dendrites and promote further dendrite branch growth in tectal neurons [71]. Conversely, blocking synapse maturation by interfering with AMPA receptor trafficking into synapses reduces dendritic arbor elaboration and completely blocks the visual stimulation-induced increase in dendritic arbor growth [52]. Therefore, the visual stimulation-induced increase in synapse number and strength [17] appears to stabilize newly extended dendritic branches. The failure of neurons with decreased insulin receptor signaling to increase their growth rate in response to visual stimulation could be a result of their low synapse density. One potential mechanism by which a lower synapse density could affect experience-dependent structural plasticity is that these neurons do not form and maintain synapses on newly added branches, and they are consequently retracted. The alternative, but not mutually exclusive, mechanism is based on the fact that, in these experiments, we transfected single tectal neurons within an otherwise normal optic tectum. Therefore, while surrounding tectal cells, which have twice the synapse density of the dnIR-expressing neurons, respond to visual stimulation normally and can increase their synapses and promote dendritic growth, the single dnIR-expressing neuron, which responds to visual inputs very weakly, may not be able to compete with normal neighboring tectal neurons for retinal inputs. Consequently, this might lead to branch length retraction and branch loss in the insulin receptor signaling deficient neurons.

Unexpectedly, we found that dnIR-expressing neurons can still elaborate their dendrites over a period of several days even when synapse density is low during early development. A similar observation was reported with manipulation of levels of the neurotrophin brain-derived neurotrophic factor, which significantly changed synapse number but not dendritic arbor morphology [147]. In the case of insulin receptor signaling where experience-dependent structural plasticity is decreased when assayed over a period of 4 hours, these daily imaging data suggest that under conditions of decreased synaptic input, alternative mechanisms participate in dendritic arbor growth control.

### Insulin receptor signaling and neurological diseases

Emergent evidence suggests an association of insulin receptor signaling with several neurological disorders. Although the role that the insulin receptor may play in these disorders is still a puzzle, enhanced brain insulin receptor signaling has been used to treat schizophrenia patients early in the mid-20th century [148,149] and insulin sensitizing drugs are now in clinical trials for the treatment of Alzheimer's disease [150-152], highlighting its importance in both neuronal developmental and degenerative diseases.

### Neurodegenerative diseases

Reduced mRNA and protein levels have been reported in postmortem material from patients with neurodegenerative disorders, for example Alzheimer's sisease [118,153] and Parkinson's disease [119], implying a role for insulin receptor signaling in neurodegenerative diseases. Among these, Alzheimer's disease is the best-studied neurodegenerative disease with respect to insulin receptor signaling.

Alzheimer's disease, the most common brain degeneration characterized clinically by progressive decline of memory and pathologically by loss of synapses, formation of neurofibrillary tangles and neuritic plaques, has been extensively studied with respect to insulin receptor signaling. Insulin receptor signaling inhibits a key event in the formation of neurofibrillary tangles by reducing tau protein phosphorylation [154,155]. Additionally, insulin receptor signaling prevents plaque formation by modulating amyloid β (Aβ) release [156] and degradation [157-160].

Although tangle formation and amyloid deposits are useful diagnostic markers, synapse loss is more robustly correlated with cognitive deficits than any other pathological lesion observable in Alzheimer's patients [161-164]. Progressive accumulation and toxicity of Aβ oligomers is the leading hypothesis for etiology of Alzheimer's disease [163]. Interestingly, the Aβ oligomer induces glutamatergic synapse loss [165,166], which in addition to cholinergic synapses seems to be most severely affected in Alzheimer's disease patients [167,168].

Furthermore, increasing evidence shows that Aβ binds to the insulin receptor, decreases the relative amount of insulin receptor in the dendritic compartment, and causes neuronal oxidative stress and loss of spines [153,169-171]. Intracellularly, Aβ is reported to block insulin receptor signaling by reducing Akt activation and eliminating its neuroprotective benefit [172,173]. Our data suggesting that insulin receptor signaling is required to maintain synapses are consistent with the model that Aβ leads to loss of synapses by directly interacting with the insulin receptor and interfering with insulin receptor signaling. Our data further support the idea that synapse loss resulting from reduced insulin receptor signaling will decrease experience-dependent structural plasticity and ultimately lead to deficits in circuit function, including information processing and integration. By contrast, reduced IGF-1 receptor function also reportedly decreases Aβ toxicity and ameliorates neuronal/synaptic loss in animal models of Alzheimer's disease [174,175]. The seemingly opposite outcomes from decreased insulin receptor and IGF-1 receptor signaling implies that either they initiate different pathways or they share the same signaling pathway but bi-directionally regulate Aβ toxicity and synaptic loss in Alzheimer's disease.

### Neuronal developmental disorders

Several neuronal developmental disorders are thought to be associated with insulin receptor signaling malfunction. For instance, schizophrenia is a chronic neurodevelopmental disorder that affects approximately 1.1% of the US population, and decreased insulin receptor protein and activity and altered downstream signaling have been reported in post-mortem schizophrenia patients [121]. Although the underlying mechanism is poorly understood, insulin treatment of schizophrenic patients was initiated during the 1930s and reportedly gives effective clinical results, [148,149]. Surprisingly, schizophrenia and Alzheimer's disease share some early pathological hallmarks, such as impaired synaptic connectivity [176-178] and abnormal dendritic structure [179,180], that eventually result in impaired circuit function and aberrant cognitive behavior.

Another example is tuberous sclerosis (TSC), a genetic disorder resulting from mutation in one of the two tumor suppressor genes, *TSC1 *or *TSC2*, that often give rise to multiple neurological deficits such as epilepsy, mental retardation and autism. Interestingly, loss of TSC function decreases synaptic transmission and alters spine morphology through the mTOR pathway, which overlaps with insulin receptor signaling [181]. One potential etiology for TSC-related neuronal deficits could be their ability to negatively regulate insulin receptor signaling in the brain as reported in the fly and in mammalian cell lines [182,183].

It is now recognized that molecules that regulate aging can also affect early neuronal development. For example, cyclin-dependent kinase 5, which plays roles in neuronal migration in the developing CNS, is also involved in the pathology of Alzheimer's disease [184]. Insulin receptor signaling, therefore, might participate at both ends of the story: early development as well as later neurodegenerative diseases.

## Perspectives

Accumulating data support the idea that insulin receptor signaling plays a prominent role in both structural and functional aspects of circuit development. The detailed cellular and molecular mechanisms by which insulin receptor signaling control synaptic function and dendritic structure are still to be determined. Besides the role of insulin receptor signaling in circuit formation, insulin receptor signaling has been linked to several neurological disorders. Whether failures in synaptic function and dendritic structure caused by decreased insulin receptor signaling contribute to brain diseases later in life is an important issue to address.

### Synapse formation or maintenance?

Precise synaptic connectivity is required for normal brain function, yet synaptic connectivity is dynamic as a result of constant synapse formation and elimination. Therefore, the reduced synapse number seen in dnIR-expressing neurons could be due to a function of insulin receptor signaling in synapse formation or synapse maintenance. If insulin receptor signaling promotes synapse formation, disrupting insulin receptor signaling should cause a shift in average maturity of the synapse population because dnIR expression would block new synapse formation but not maturation of existing synapses; however, postsynaptic AMPA/NMDA ratios or presynaptic maturation indexes were not different in dnIR-expressing neurons compared to controls. Furthermore, our ultrastructural observations indicate that the synapses onto dnIR-expressing neurons have similar maturation indices as controls. Finally, we find that the fraction of silent synapses is not altered in dnIR-expressing neurons compared to controls. These three pieces of evidence suggest that insulin receptor signaling is not required for synapse formation and is, therefore, more likely to regulate synapse connectivity through synapse maintenance. Collectively, these results indicate that synapse maturation and the balance of synapse formation and elimination could be separately regulated *in vivo*, and that insulin receptor signaling has an impact specifically on the synapse numbers by regulating synapse elimination. Moderate expression of PSD-95 has been used as an *in vivo *synaptic marker without significantly affecting synaptic density in *Xenopus *tadpoles and other vertebrates [147,185]. Therefore, it would be interesting to perform *in vivo *time-lapse imaging to monitor synapse dynamics by tracking fluorescently tagged PSD-95 puncta in optic tectal cell dendrites. Detailed analysis of synapse behaviors - for example, to determine numbers of added, stable and lost synapses in dnIR- or GFP-transfected neurons - would provide a direct test of the hypothesis and could elucidate the cellular mechanism of insulin receptor signaling in regulating synapse connectivity.

### Endogenous ligand and receptor composition

Insulin is thought to be the primary ligand for the insulin receptor; however, IGF-2 also reportedly binds the homodimer of the insulin receptor splice variant in the brain [95]. Moreover, the discovery that the insulin receptor and IGF-1 receptor heterodimerize expands the potential ligands for insulin receptor heterodimers in the brain to include insulin, IGF-2, IGF-1 and possibly others [86]. Several potential ligands - for example, mammalian insulin and nematode insulin and IGFs - have been reported to affect synaptic transmission and plasticity, dendrite structure, whole animal lifespan and behaviors in various model systems [101,105-107,186-189]. The identity of the primary ligand(s) that activate insulin receptor signaling and regulate synapse number, where the ligands are found in the brain and how are they regulated are all important questions requiring further exploration.

At the receptor level, it is important to investigate the composition of the receptor dimer since it determines the specificity and affinity of the ligand(s) and might initiate distinct downstream signaling pathways. Our strategy of using dnIR expression can potentially disrupt three types of receptor signaling according to the receptor composition: the insulin receptor homodimer; the insulin receptor-IGF-1 receptor heterodimer; and the IGF-1 receptor homodimer. It is interesting to note that when antisense morpholino oligonucleotides were used to specifically knockdown insulin receptor but not IGF-1 receptor, morpholino-transfected neurons show a similar deficit in visual responses recorded *in vivo *compared to dnIR-expressing neurons. This result indicates that the insulin receptor, instead of the IGF-1 receptor, plays major roles in visual circuit function. Nevertheless, whether the insulin receptor executes its function through the insulin receptor homodimer or the insulin receptor/IGF-1 receptor heterodimer is still an open question. Traditional co-immunoprecipitation of the insulin receptor dimers from brain lysate might help in deciphering the receptor composition if one can develop specific antibodies to differentiate these two structurally similar receptors. Alternatively, molecular tools - for example, morpholino or RNA interference - to specifically knockdown the insulin receptor, the IGF-1 receptor alone, or both together may provide further insight.

### Molecular mechanisms

The decrease in insulin receptor signaling by dnIR expression affects visual responses in tectal neurons to the same extent as morpholino-mediated knockdown of insulin receptor protein, indicating that kinase activity of the insulin receptor plays a major role in insulin receptor function. What are the downstream cascades activated by insulin receptor kinase activity in the CNS? Studies in peripheral tissues have demonstrated that MAPK or Akt are major pathways downstream of the insulin receptor [88]. Whether MAPK or Akt pathways underlie insulin receptor-mediated circuit development needs to be further explored. In addition to these general pathways, some molecules appear to be more specific to insulin receptor signaling, for example, IRSs [190]. As mentioned before, IRSp53 is a great candidate to execute insulin receptor function at excitatory synapses by regulating the actin cytoskeleton through a pathway that requires its coupling with activated Rho GTPase [77,140,141]. Whether this effect on actin cytoskeleton originates from insulin receptor signaling would be interesting to know. Recently, the phosphorylation sites of IRSp53 that specifically respond to insulin receptor signaling have been discovered [191]. Mutations of these sites would allow us to understand the interplay between the insulin receptor, IRSp53 and RhoGTPases in the structural aspects of circuit development.

### Neurological disorders

Accumulating data suggest a potential link between insulin receptor signaling and several neurological disorders. As mentioned above, enhanced insulin receptor signaling has been one strategy for clinical treatments for patients with Alzheimer's disease [150-152] and schizophrenia [148,149], although the underlying mechanism is not clear. One common pathological hallmark for Alzheimer's disease and schizophrenia is the problem in circuit function as a result of reduced synaptic connectivity [176-178,192]. The discovery of a crucial role for the insulin receptor in synapse maintenance and circuit function suggests a cellular mechanism to illustrate how impaired insulin receptor signaling may contribute to neurological disorders. Nevertheless, improved treatments and additional research are needed to understand the detailed molecular mechanism by which the insulin receptor affects synapse loss or function of brain circuits. A transgenic model system in which insulin receptor levels or signaling could be controlled with spatial and temporal resolution would be beneficial in exploring the detailed mechanisms at the molecular level and the pathogenesis at the behavioral level. Since whole system knockouts of insulin receptor in mice are lethal [193], conditional knockouts will be required. In fact, neuron specific insulin receptor knockout mice that are viable have been developed and they show decreased phosphorylation of Akt and glycogen synthase kinase 3 beta. Interestingly, glycogen synthase kinase 3 beta is again highly associated with Alzheimer's disease [155,194] and schizophrenia [195]. Further research on this type of transgenic system will provide insight into the physiological function of the insulin receptor in the development of the normal brain as well as the etiology of neurological diseases.

## Abbreviations

Aβ: amyloid β; AMPA: α-amino-3-hydroxy-5-methyl-4-isoxazole propionic acid; CNS: central nervous system; dnIR: dominant negative insulin receptor; GABA: γ-aminobutyric acid; GFP: green fluorescent protein; GluR: glutamate receptor; IGF: insulin-like growth factor; IR: insulin receptor; IRS: insulin receptor substrate; MAPK: mitogen-activated protein kinase; mEPSC: miniature excitatory postsynaptic currents; mTOR: mammalian target of rapamycin; NMDA: N-methyl-D-aspartate; PI3K: phosphoinositide-3 kinase; PSD: postsynaptic density; PSD-95: postsynaptic density protein-95; TSC: tuberous sclerosis.

## Competing interests

The authors declare that they have no competing interests.

## Authors' contributions

S-LC and HTC discussed the content and wrote the manuscript.
